# Different dimensions of oral health literacy and tooth avulsion management: pre-post study with schoolteachers

**DOI:** 10.1590/1807-3107bor-2025.vol39.123

**Published:** 2025-11-17

**Authors:** Fabio Anevan Ubiski FAGUNDES, Anny Caroline SCHADE, Vinicius Felipe Oliveira DIAS, Fabian Calixto FRAIZ, Fernanda de Morais FERREIRA, Luciana Reichert da Silva ASSUNÇÃO

**Affiliations:** (a) Universidade Federal do Rio de Janeiro – UFRJ, School of Dentistry, Department of Pediatric Dentistry and Orthodontics, Rio de Janeiro, RJ, Brazil.; (b) Universidade Federal do Paraná – UFPR, School of Dentistry, Department of Stomatology, Curitiba, PR, Brazil.; (c) Universidade Federal de Minas Gerais, School of Dentistry, Department of Oral Health for Children and Adolescents, Belo Horizonte, MG, Brazil.

**Keywords:** Health, Attitude, Health Literacy, Tooth Avulsion, School Teachers

## Abstract

Oral health literacy (OHL) may play a crucial role for managing traumatic dental injuries. In this pre-post study, the effect of the different dimensions of OHL on the efficacy of using an information leaflet (IL for managing permanent tooth avulsion ) was assessed in elementary schoolteachers. Functional OHL was measured using the Brazilian version of the Rapid Estimate of Adult Literacy in Dentistry (BREALD-30), while interactive OHL was assessed with the Oral Health Literacy Adult Questionnaire (BOHL-AQ). Knowledge scores (KS) on avulsion management were collected at three intervals: before reading the IL (baseline), immediately post-intervention (Phase I), and 60 days post-intervention (Phase II). The effect on OHL was assessed by comparing KS means between Phase I/Baseline (acquisition) and Phases II/I (retention). Participants with high OHL/BOHL had a greater difference in mean KS values between Phase I/baseline, while those with high OHL/BREALD-30 showed a smaller difference between phases II/I. Different dimensions of OHL impacted the effectiveness of an educational intervention on managing permanent tooth avulsion with use of a leaflet.

## Introduction

Tooth avulsion is considered one of the most serious traumatic dental injuries (TDI),^1^ and adequate and immediate management is a decisive factor in the prognosis of reimplantation of the tooth affected.^2^ Its prognosis depends on the extra-alveolar time, storage medium, periodontal ligament injury, root development and how the avulsion injury was treated.^1^ Ideally, the tooth should be repositioned in its alveolus immediately or within a maximum of fifteen minutes after the accident.^1^

Schools are some one of the environments with the highest occurrence of TDI.^3^ Therefore, it is essential for teachers to have adequate knowledge about the management of these situations to help with the care of children affected.^4^However, previous studies have demonstrated that teachers of schoolchildren have insufficient knowledge about the management of different types of dental trauma.^5-7^ A recent systematic review and meta-analysis assessed knowledge about management of dental trauma by teachers of schoolchildren and observed low levels of both knowledge and self-confidence for decision-making in the face of these occurrences.^8^

With the purpose of improving teachers’ knowledge relative to the management of TDI, educational interventions have been proposed ^9-12^ and include different methods such as leaflets^13^, presentations^11^ and educational posters.^12^ The studies have been unanimous in confirming an improvement in knowledge after the interventions.^9-13^

Several factors can influence the knowledge of health outcomes, and in recent years, researchers have focused efforts on understanding the relationship between professional-patient communication strategies and health literacy.^14^Health literacy is considered multidimensional and encompasses different competences that include basic reading and writing skills for everyday life (basic/functional literacy), skills that allow individuals to extract information and apply it to changing life circumstances (communicative/interactive literacy) and more advanced competences that allow the critical analysis of information (critical illiteracy).^15^ These dimensions can be extrapolated to oral health literacy (OHL) defined as the degree to which individuals are able to obtain, process, understand oral health information and make appropriate decisions in health.^15^

A recent study has observed that parents and caregivers with lower OHL levels in the functional and interactive dimensions, showed less understanding after reading an educational leaflet.^16^ These results pointed to the need for identifying the different levels of OHL, and their modifying effect on the efficacy of intervention conducted for managing dental trauma. This particularly applies to dental avulsion that requires immediate and appropriate action when the incident occurs. This justification gains interest when a more educated population, such as schoolteachers, is considered.

The null hypothesis of this investigation was that there would be no difference in the effectiveness of an information leaflet on managing permanent tooth avulsion considering different OHL dimensions. Thus, the aim of this pre-post study was to investigate the modifying effect of different dimensions of OHL on the effectiveness of an educational intervention in the management of permanent tooth avulsion, applied to elementary school teachers.

## Methods

### Ethical aspect

The study was assessed and approved by the Ethics Committee for Research with Human Beings of the Federal University of Paraná (process number 04687118.6.0000.0102, opinion number 3.175.223). All the participants signed the term of free and informed consent.

### Study population and sampling

The study population was composed of elementary schoolteachers of children between six and 12 years of age enrolled in public schools in the city of Pinhais, in the south of Brazil. Teachers over 18 years of age and native speakers of Brazilian Portuguese were included. Data was collected in the period from April 2019 to March 2020.

To calculate the sample size, the correlation coefficient test was used considering the knowledge score (KS) and the oral health literacy (OHL) score evaluated by two different instruments: The Brazilian Rapid Estimate of Adult Literacy in Dentistry (BREALD-30) and Brazilian Oral Health Literacy Adult Questionnaire (BOHL-AQ) obtained in a pilot study. The minimum sample was calculated considering an alpha error of 5% and test power of 80%.

After the linear correlation analysis, a correlation coefficient of 0.302 was identified between KS and the OHL/BREALD-30 score, resulting in a minimum sample of 85 teachers. The correlation coefficient between KS and the OHL/BOHL-AQ score was 0.226, resulting in a minimum sample of 152 participants. As the result obtained by the BOHL-AQ instrument resulted in a higher number for the minimum sample (n=152), this parameter was considered for the minimum sample. After adding 30% for possible losses, the total was the maximum sample of 200 participants.

Participants were selected by simple random sampling, without replacement, using a list. Sample calculation and randomization of participants were performed using the Biostat Program version 5.3 (Mamirauá Institute, Brazil).

### Pilot study

In the pilot study, the questionnaire formulated specifically for this research was applied to 12 elementary school teachers in a municipal school in the same municipality as that of the main study. The aim was to verify the applicability of the instrument for obtaining data that would allow the research questions of the study to be answered. Those who participated in this phase did not participate in the final sample. Some words and questions in the questionnaire were reformulated in order to improve participants’ understanding. The study was conducted in April 2019.

### Oral health literacy

To assess the level of literacy in oral health, two instruments were applied (REALD-30^17^ and OHL-AQ^18^) in their versions that were translated and validated for Brazilian Portuguese: the Brazilian Rapid Estimate of Adult Literacy in Dentistry (BREALD-30)^19^ and the Brazilian Oral Health Literacy - Adult Questionnaire (BOHL-AQ),^20^ at the baseline of the study.

The BREALD-30 is an instrument that assesses the functional dimension of OHL and consists of a list of 30 words related to oral health arranged in increasing order of difficulty, which the participant must read aloud to the interviewer. For each word read correctly, one point is added to the participant’s score and for words read incorrectly, a score of 0 is recorded. Therefore, the score can vary between 0 (lowest literacy) and 30 (highest literacy).^19^ Prior to conducting the tests, an evaluator (FAUF) was trained according to criteria established by Vilella et al (2016).^23^ Reliability of the method was analyzed using the Kappa coefficients, in which the right and wrong answers for each word in the instrument were considered, and by the intraclass correlation test (ICC) considering the total score of each video. The inter- and intra-examiner Kappa values were 0.911 and 0.937, respectively, and the inter- and intra-examiner ICC values, 0.981 and 0.996, respectively.

The BOHL-AQ involves broader dimensions of OHL and therefore includes four sections: reading and comprehension, numeracy, listening and decision making. This instrument consists of 17 items divided into 14 questions that address concepts of reading comprehension (reading and knowledge skills), numeracy (reading, writing and calculation skills), listening (listening, reading, writing, calculation and communication skills), appropriate decision-making, and conceptual knowledge (reading, comprehension, and decision-making skills). Correct answers received a score of 1 (one) and incorrect answers, 0 (zero). The total score varied between 0 and 17, with higher scores being observed in participants with higher OHL.^20^

### Questionnaires

To assess knowledge relative to the management of permanent tooth avulsion, a questionnaire was constructed specifically for this study. The knowledge score (KS) on the conduct of tooth avulsion was obtained by means of six statements (Table 1) with answers arranged on a three-point Likert scale ranging from: “I agree”, “I neither agree nor disagree” and “I disagree”. There was still another response option “I don’t know”. For each correct answer, a score of 1 (one) was assigned, and for incorrect answers, answers such as “I neither agree nor disagree” and “I don’t know”, a score of 0 (zero) was assigned. Therefore, the final scores ranged between 0 (zero) and 6 (six). Socioedemographic information was gathered by means of a questionnaire previously tested in a pilot study. The sociodemographic variables included age, sex, professional formation, and time engaged in the profession.

**Table 1:** Statements used to assess the knowledge score in the three phases of the study.

Statements	Correct abswer
After washing the tooth with water I must scrape the dirt from the root.	Do not agree
I must not hold the tooth by the root.	Agree
I can put the tooth back in its place after cleaning it (the tooth).	Agree
If you are going to take the tooth to the dentist, it is best to place the tooth in a glass of tap water.	Do not agree
I can also place the tooth between the cheek and the gum.	Agree
All of this must be done within a time of up to 6 hours to arrive at the dentist.	Do not agree

### Educational intervention and phases of the study

The educational intervention consisted of reading a leaflet about conducting a case of tooth avulsion. This leaflet was created by the International Association for Dental Traumatology (IADT) and is available online.^21^ The leaflet used was the version translated into Brazilian Portuguese by the Brazilian Society of Dental Traumatology (SBTD).^22^

**Figure 1:**
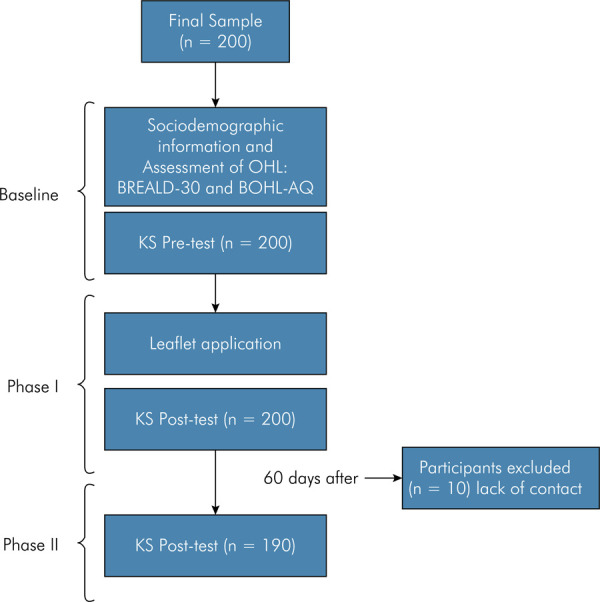
Flowchart that describes phases of the study.

Knowledge was assessed at three different time intervals: before an educational intervention (Baseline), immediately after (Phase I) and 60 days after the educational intervention (Phase II). (Figure 1)

### Statistical analysis

The descriptive values of this study were assigned according to measures of central tendency, variability for numerical variables, and according to their absolute and relative values for categorical variables. OHL and KS Scores were submitted to the Kolmogorov Smirnov normality test. Since p < 0.05, non-parametric tests were used for inferential analyses.

Correlations between KS and OHL/BREALD-30 and OHL/BOHL-AQ scores, were analyzed by using Spearman’s correlation test.

Participants were categorized into high and low OHL considering the median value of the BREALD-30 scores (high: score≥28; low: score ≤ 27) and BOHL-AQ (high: score ≥ 15; low: score ≤ 14). For comparison of KS mean values within the same group in the three phases of the study, the Friedman non-parametric test was used. In case of significance, the Wilcoxon test was performed to analyze significance in dependent groups of two.

To evaluate the modifying effect of OHL on the effectiveness of the educational intervention, the difference in KS means between Phases I and baseline (acquisition of information) and between Phases II and Phase I (retention of information) were considered. Values were compared considering the different groups (high and low OHL) and analyzed using the non-parametric U Mann-Whitney test.

All statistical analyses were performed with the use of the STATA program 12.0 (StataCorp LP, College Station, USA). The level of significance adopted for all the analyses was 5%.

## Results

A total of 200 subjects participated in baseline and Phase I of the study. There was no exclusion according to the eligibility criteria and no refusals to participate in this study. A total of 190 teachers participated in Phase II of the study (follow-up test). The reason for the losses was the lack of contact with 10 participants (9.5%).

Table 2 presents the characteristics of the population at baseline and in Phase I of the study. The mean age was 41.3 years (standard deviation [SD] = 9.3), and the majority of respondents were female (98%). As regards teacher training, the majority of participants had a background in Pedagogy (96.5%), with an average of 16.47 years in the profession (SD = 9.648). The OHL/BREAD-30 had a median score of 28 (minimum 19, maximum 30), while the OHL/BOHL-AQ had a median score of 15 (minimum 8, maximum 17).

**Table 2:** Characteristics of the population in Baseline and Phase I (n = 200).

Variables	
Age in years - mean (SD)	41.34 (9.315)
Sex - n (%)	
Female	196 (98.0)
Male	4 (2.0)
Professional background - n (%)	
Pedagogy	193 (96.5)
Physical education	2 (1.0)
Others	5 (2.5)
Time engaged in profession (in years) - mean (SD)	16.47 (9.648)
BREALD-30	
Median (minimum- maximum)	28 (19–30)
BOHL-AQ	
Median (minimum- maximum)	15 (8–17)

Frequency values lower than 200 resulted from absence of data for variable.

Table 3 shows the results of the Spearman correlation test when comparing the KS values evaluated in the three time intervals and the OHL scores of the BREALD and OHL-AQ instruments, as well as in each domain of the BOHL-AQ. The results showed that there was a positive and significant correlation for the knowledge score at baseline only for the domain “Listening” of the BOHL-AQ instrument. In Phase I, there was a positive and significant correlation between the KS score and the OHL score in the two instruments and for all BOHL-AQ domains. Similarly, there was a positive and significant correlation between the Phase II knowledge score and the other OHL scores, with the exception of the “Listening” domain of the BOHL-AQ instrument.

**Table 3:** Correlation between knowledge score assessed in Baseline, Phases I and II and OHL scores, assessed by BREALD-30 and BOHL-AQ.

Variables	r_s_ **	P*
Knowledge score (Baseline )		
BREALD-30	0.125	0.079
BOHL-AQ (Total)	0.103	0.146
Domains- BOHL-AQ		
Reading comprehension	0.088	0.213
Numeracy	0.028	0.689
Listening	0.142	**0.045**
Decision making and conceptual knowledge	-0.015	0.833
Knowledge score (Phase I)		
BREALD-30	0.170	**0.016**
BOHL-AQ (Total)	0.244	**0.001**
Domains- BOHL-AQ		
Reading comprehension	0.148	**0.036**
Numeracy	0.161	**0.023**
Listening	0.162	**0.022**
Decision making and conceptual knowledge	0.161	**0.023**
Knowledge Score (Phase II)		
BREALD-30	0.368	**<0.001**
BOHL-AQ (Total)	0.285	**<0.001**
Domains- BOHL-AQ		
Reading comprehension	0.229	**0.004**
Numeracy	0.186	**0.019**
Listening	0.129	0.105
Decision making and	0.172	**0.030**

BREALD-30: Brazilian Rapid Estimate of Adult Literacy in Dentistry; BOHL-AQ: Brazilian Oral Health Literacy- Adult Questionnaire. *Spearman correlation test; **Spearman correlation coefficient. Statistically significant values are highlighted in bold font.

Means followed by different letters indicate statistically significant differences for intragroup comparison between the different phases of the study (p < 0.001, Wilcoxon test for comparisons in two dependent groups).

**Figure 2:**
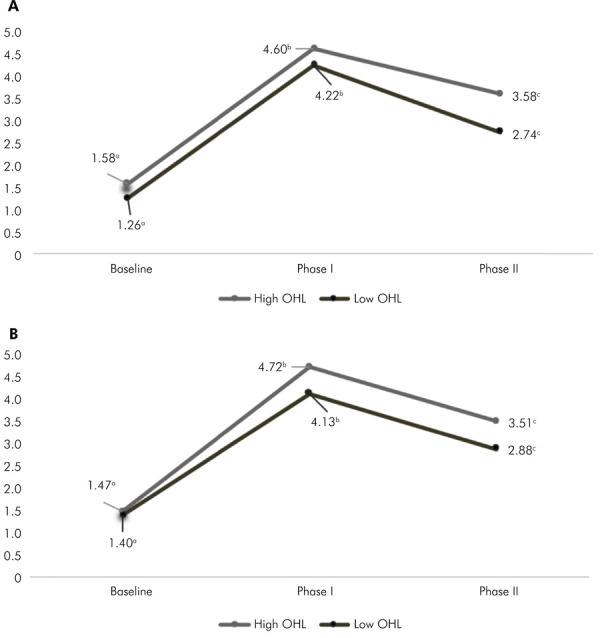
Knowledge score means at the three times evaluated periods (Baseline/Phase I/ Phase II) according to the levels of literacy assessed by: a) BREALD-30 and b) BOHL-AQ in the intra-group comparison.

Of the total of 200 participants at baseline, 110 (55.0%) presented a high level of oral health literacy for BREALD-30 and 102 (51.0%) for BOHL-AQ. Figure 2 shows the comparison of mean KS values within the same group in the three phases of the study, stratified by OHL levels for OHL/BREALD-30 (Figure 2a) and OHL/BOHL-AQ (Figure 2b). There was a significant increase in KS scores immediately after the intervention, in individuals with high and low OHL assessed by both instruments. In contrast, two months after the intervention, a significant decrease in KS was observed in the groups with high and low OHL, assessed by both instruments.

Table 4 shows the difference in mean KS values between Phases I and baseline and between Phases II and Phase I according to literacy level. Participants with high OHL assessed by BOHL-AQ showed a greater difference in mean values of KS in Phase I and baseline (p = 0.020), while individuals with high OHL assessed by BREALD-30 showed a smaller difference in mean values in phases II/I (p =0.002).

**Table 4:** Comparison of the difference between the means of KS in Phases I/Baseline and Phases II/I in individuals with high and low OHL evaluated by BREALD-30 and BOHL-AQ.

Variables	High OHL	Low OHL	p-value*
Phases I/Baseline	KS Mean difference (SD)	KS Mean difference (SD)	
BREALD-30	3.018 (1.675)	2.967 (1.561)	0.654
BOHL-AQ	3.245 (1.619)	2.735 (1.590)	**0.020**
Phases II/ I	Mean difference (SD)	Mean difference (SD)	
BREALD-30	-0.966 (1.401)	-1.529 (1.348)	**0.002**
BOHL-AQ	-1.790 (1.355)	-1.253 (1.462)	0.647

SD: standard deviation, *U Mann-Whitney test. Statistically significant values are highlighted in bold font.

## Discussion

In the literature, this study is one of the few that portrays the behavior of different dimensions of the OHL relative to the effectiveness of an educational intervention. The results of this study showed that different dimensions of OHL were able to modify the effect of an educational intervention in the management of permanent tooth avulsion in a sample of elementary school teachers. Although the OHL values, particularly the BREALD-30, were higher when compared with those of other populations in the same region of Brazil,^16,23^ this justifies the importance of identifying the impact of literacy on oral health interventions even in populations with higher levels of education.

Moreover, although the educational level is an important predictor of OHL, they are two different concepts.^14^ Literacy involves a great deal more than years of formal studies, but also includes previous experiences, individual characteristics, in addition to the individual’s own state of health.^24^Thus, the years of schooling completed may not fully estimate personal skills with regard to appropriate health decisions, and there may be other factors involved in this process.^14^ Recently, interest has increased in the interactive and critical dimensions of OHL, which involve cognitive and social skills needed to critically assess the applicability of health information to personal situations.^25^ In this sense, the relationship between education and OHL may vary throughout different populations, particularly considering social inequalities and disparities in access to health information.^26^

The efficacy of the educational intervention displayed different patterns when accounting for the two assessment instruments and comparing individuals with high and low levels of OHL. While the functional dimension played a pivotal role in sustaining knowledge retention over time, the comprehensive dimensions assessed by the BOHL-AQ proved more salient during the initial knowledge acquisition phase. Consequently, participants with elevated BOHL-AQ showed a greater capacity to read and comprehend the leaflet content, resulting in a more assured application of the elements included. It is noteworthy that the multifaceted nature of BOHL-AQ’, covering various aspects of literacy, including reading, comprehension, numeracy, and decision-making, may elucidate its immediate impact on post-leaflet reading.

The study findings also indicated that individuals with higher functional OHL levels evaluated by BREALD-30, exhibited a less substantial decline in acquired knowledge after two months of interacting with the educational material when compared with those with low OHL. A randomized controlled study conducted with Brazilian pregnant women observed that participants with higher levels of OHL, assessed by the BREALD-30, showed better performance in reading comprehension of a leaflet that addressed information on children’s oral health. in this same study it was observed that pregnant women with higher OHL had greater retention of knowledge at 60 days after the intervention.^27^

Taking into consideration the correlation between the knowledge score and the OHL values of each instrument, only the “listening” domain of the BOHL-AQ remained associated with KS at baseline. This domain represents adequate communication skills and is related to a greater capacity for understanding instructions on oral health practices, including preventive and maintenance care.^18^ In this study, although no interference of previous information was shown with regard to the management of the avulsed permanent tooth, it is possible that individuals with greater ability to understand health information may have had better knowledge about the subject prior to receiving the guidelines.

Unlike the baseline results, OHL score values assessed by both instruments exhibited a positive correlation with KS in Phases I and II. These findings underscore the impact of functional dimensions (BREALD-30) and broader dimensions (BOHL-AQ) on initial knowledge acquisition and its retention over time. In a previous study using the same educational leaflet among a sample of parents and caregivers of children, participants with high OHL, evaluated by means of functional and interactive dimensions, reported a superior understanding of the information and a greater perceived capability in performing the tasks outlined in the leaflet, when compared with those with low OHL^16^.

When comparing the mean KS values at different evaluation time intervals in intragroup analyses, there was an initial increase in knowledge immediately after the intervention. However, two months after application of the educational leaflet, a decline in knowledge was observed in both groups, as indicated by both instruments. These findings underscore the importance of ongoing education, irrespective of the initial OHL levels, consistent with findings of previous studies.^27,28^ In addition, it was possible to observe that individuals with lower OHL levels in the functional dimension exhibited a more pronounced loss of information in the long term. This highlights the significance of providing enhanced attention to this particular group when devising educational strategies.

Individuals with low OHL may benefit from multiple approaches to support their learning and retention of essential information.^29^ Multimodal strategies leverage various methods to enhance learning by engaging different sensory channels and accommodating diverse learning preferences.^29^These approaches often integrate visual aids, interactive sessions, and hands-on demonstrations, each contributing uniquely to knowledge acquisition.^29^By combining these methods, a more comprehensive and effective learning environment can be created, potentially improving comprehension and retention in dental trauma knowledge.^30^Future interventions should consider incorporating multimodal approaches to address the varied needs of learners, particularly those with low functional OHL. Such strategies could play a crucial role in enhancing the overall effectiveness of educational programs and promoting long-term information retention.

Although the present research is one of the few that has presented an interventional proposal in the context of the OHL, its follow-up time was a limitation. Therefore, the recommendation is that additional studies, with assessments carried out over extended durations, should be conducted. Furthermore, generalizations must be made with caution as the characteristics of the magisterium may differ between different regions. As with any pre-post study design, the absence of a comparison group limited precision, rendering it more susceptible to internal validity bias.^31^

## Conclusion

Different dimensions of OHL influenced the effectiveness of an educational intervention on the management of permanent tooth avulsion using a leaflet, throughout various evaluation periods. Therefore, health education strategies—even when targeting individuals with higher education levels, such as teachers, should take into account the varying levels and dimensions of OHL

## Data Availability

After publication the data will be available on demand to the authors - condition justified in the manuscript.
